# Charge transfer through single molecule contacts: How reliable are rate descriptions?

**DOI:** 10.3762/bjnano.2.47

**Published:** 2011-08-03

**Authors:** Denis Kast, L Kecke, J Ankerhold

**Affiliations:** 1 Universität Ulm, Institut für Theoretische Physik, Albert-Einstein-Allee 11, 89069 Ulm, Germany

**Keywords:** inelastic charge transfer, molecular contacts, nonequilibrium distributions, numerical simulations, rate equations

## Abstract

**Background:** The trend for the fabrication of electrical circuits with nanoscale dimensions has led to impressive progress in the field of molecular electronics in the last decade. However, a theoretical description of molecular contacts as the building blocks of future devices is challenging, as it has to combine the properties of Fermi liquids in the leads with charge and phonon degrees of freedom on the molecule. Outside of ab initio schemes for specific set-ups, generic models reveal the characteristics of transport processes. Particularly appealing are descriptions based on transfer rates successfully used in other contexts such as mesoscopic physics and intramolecular electron transfer. However, a detailed analysis of this scheme in comparison with numerically exact solutions is still elusive.

**Results:** We show that a formulation in terms of transfer rates provides a quantitatively accurate description even in domains of parameter space where strictly it is expected to fail, e.g., at lower temperatures. Typically, intramolecular phonons are distributed according to a voltage driven steady state that can only roughly be captured by a thermal distribution with an effective elevated temperature (heating). An extension of a master equation for the charge–phonon complex, to effectively include the impact of off-diagonal elements of the reduced density matrix, provides very accurate solutions even for stronger electron–phonon coupling.

**Conclusion:** Rate descriptions and master equations offer a versatile model to describe and understand charge transfer processes through molecular junctions. Such methods are computationally orders of magnitude less expensive than elaborate numerical simulations that, however, provide exact solutions as benchmarks. Adjustable parameters obtained, e.g., from ab initio calculations allow for the treatment of various realizations. Even though not as rigorously formulated as, e.g., nonequilibrium Green’s function methods, they are conceptually simpler, more flexible for extensions, and from a practical point of view provide accurate results as long as strong quantum correlations do not modify the properties of the relevant subunits substantially.

## Introduction

Electrical devices on the nanoscale have received substantial interest in the last decade [1]. Impressive progress has been achieved in contacting single molecules or molecular aggregates with conducting or even superconducting metallic leads [2–3]. The objective is to exploit nonlinear transport properties of molecular junctions as the elementary units for a future molecular electronics. While the initial experiments were operated at room temperature, low temperatures down to the millikelvin range, the typical regime for devices in mesoscopic solid state physics, are also accessible (see, e.g., [4–6]). This allows for detailed studies of phenomena such as inelastic charge transfer due to molecular vibrations [7–9], voltage driven conformational changes of the molecular backbone [10], Kondo physics [11], and Andreev reflections [6], to name but a few.

These developments have been accompanied by efforts to advance theoretical approaches in order to obtain an understanding of general physical processes on the one hand and to arrive at a tool to quantitatively describe and predict experimental data. For this purpose, basically two strategies have been followed. One is based on ab initio schemes that have been successfully employed for isolated molecular structures, such as, e.g., density functional theory (DFT). Combining DFT with nonequilibrium Green’s functions (NEGF) allows us to capture essential properties of junctions with specific molecular structures and geometries [2–3,12–13]. This provides insight into the electronic formations of contacted molecules and gives at least qualitatively correct results for currents and differential conductances. However, a quantitative description at the level of accuracy known from conventional mesoscopic devices still seems to be out of reach. Furthermore, these methods are not able to capture phenomena resulting from strong correlations effects, such as Kondo resonances.

Thus, an alternative route, mainly inspired by solid state methodologies, starts with simplified models that are assumed to cover the relevant physical features. The intention then is to reveal fundamental processes characteristic for molecular electronics that give a qualitative description of observations from realistic samples, but provide also the basis for a proper design of molecular junctions to exploit these processes. Information about specific molecular set-ups appears merely in the form of parameters which offer a large degree of flexibility. In general, to attack the respective many body problems, perturbative schemes have been applied, the most powerful of which are nonequilibrium Green’s functions [14–15]. However, conceptually simpler, easier to implement, and often better at revealing the physics, are treatments in terms of master or rate equations. Being approximations to the NEGF frame in certain ranges of parameters space, they sometimes lack the strictness of perturbation series, but have been extensively employed for mesoscopic devices [16] and quantitatively often provide solutions of at least similar accuracy. Roughly speaking, these schemes apply as long as quantum correlations between relevant subunits of the full compound are sufficiently weak [15]. Physically, it places charge transfer through molecular contacts in the context of inelastic charge transfer through ultrasmall metallic contacts (dynamical Coulomb blockade [17]) and in the context of solvent or vibronic mediated intramolecular charge transfer (Marcus theory) [18–20].

While rate descriptions have been developed in a variety of formulations before [21–28], the performance of such a framework in comparison with numerically exact solutions has not yet been addressed. The reason for this is simple: A numerical method that provides numerically exact data in most ranges of parameters space (temperature, coupling strength, etc.) has only very recently been successfully implemented in the form of a diagrammatic Monte Carlo approach [29]. Path integral Monte Carlo methods have been used previously for intramolecular charge transfer in complex aggregates [18–19] in a variety of situations, including correlated [30] and externally driven transfer [31] and, of particular relevance to the present work, transfer in the presence of prominent phonon modes [32].

The goal of the present work is to study a simple yet highly nontrivial set-up, namely, a molecular contact with a single molecular level coupled to a prominent vibronic mode (phonon) which itself may or may not be embedded in a bosonic heat bath. We develop rate descriptions of various complexity, place them into the context of NEGF, and compare them with exact solutions. The essence of this study is, astonishingly enough, that rate theory provides quantitatively accurate results for mean currents over a very broad range of parameter space, even in domains where they are not expected to be reliable.

## Results and Discussion

In subsection 1 we define the model and the basic ingredients for a perturbative treatment. A formulation which closely follows the *P*(*E*) theory for dynamical Coulomb blockade is discussed in subsection 2. Nonequilibrium effects in the stationary phonon distribution are analyzed in subsection 3 based on a dynamical formulation of charge and phonon degrees of freedom. The presence of a secondary bath is incorporated in subsection 4 together with an improved treatment of the molecule–lead coupling, which is exact for vanishing electron–phonon interaction. The comparison with numerically exact data and a detailed discussion is given in subsection 5.

### 1 Model

**Figure 1:**
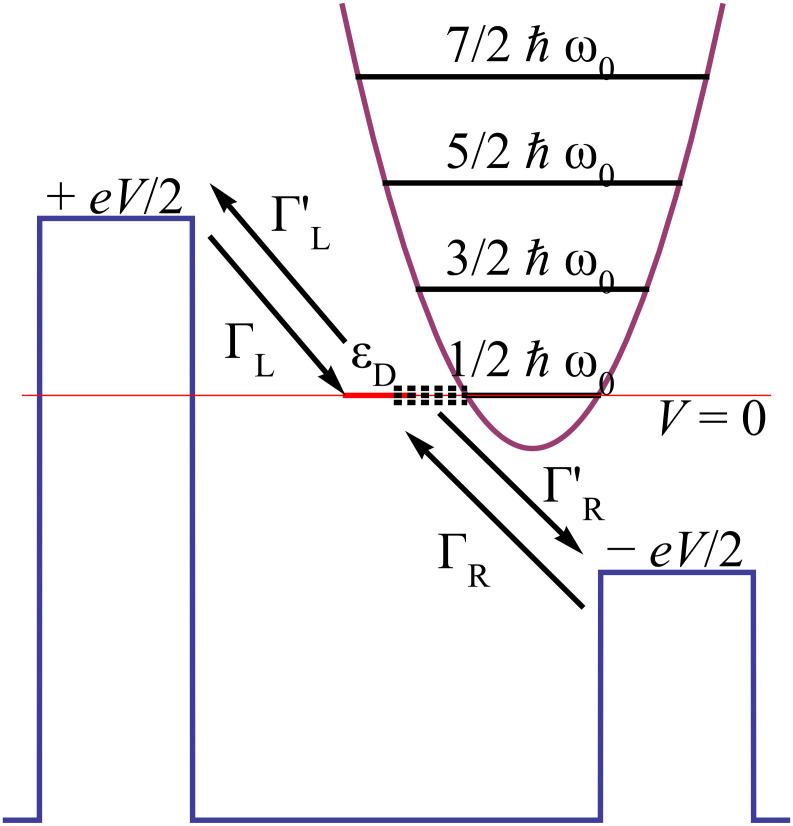
Single charge transfer through a molecular contact consisting of a single electronic level coupled to a harmonic phonon mode and contacted to metallic leads. Forward (no prime) and backward (with prime) rates are the basic ingredients for the approximate treatment, see text for details.

We start with the minimal model of a molecular contact consisting of a single electronic level (dot) coupled to fermionic reservoirs, where a prominent internal molecular phonon mode interacting with the excess charge is described by a harmonic degree of freedom (Figure 1) [15,33–34].

Neglecting spin degrees of freedom the total compound Hamiltonian is thus described by

[1]
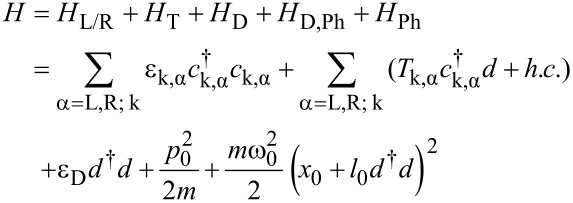


Here, the *T*_k,α_ denote tunnel couplings between dot level and reservoir α and 
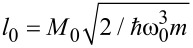
 contains the coupling *M*_0_ between excess charge and phonon mode. An external voltage *V* across the contact is applied symmetrically around the Fermi level such that ε_k,α_ = ε_0_(*k*) + *μ*_α_, with the bare electronic dispersion relation ε_0_(*k*) and chemical potentials *μ*_L_ = +*eV*/2, *μ*_R_ = −*eV*/2. Below, this model will be extended further to include the embedding of the prominent mode into a large reservoir of residual molecular and/or solvent degrees of freedom acting as a heat bath. Qualitatively, since the dot occupation *d*^†^*d* can only take the values *q* = 0 or 1, the sub-unit *H*_D_ + *H*_D,Ph_ + *H*_Ph_ describes a two state system coupled to a harmonic mode (spin–boson model [20]). Depending on the charge state of the dot the phonon mode is subject to potentials *V*_q_(*x*_0_) = (*m*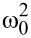/2)(*x*_0_ + *l*_0_*q*)^2^. Now, the presence of the leads acts (for finite voltages) as an external driving force alternately charging (*q* = 1) and discharging (*q* = 0) the dot, thus switching alternately between *V*_0_ and *V*_1_ for the phonon mode. The classical energy needed to reorganize the phonon is the so-called reorganization energy 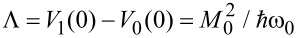. Quantum mechanically, the phonon mode may also tunnel through the energy barrier located around *x*_0_ = −*l*_0_/2 separating the minima of *V*_0,1_.

It is convenient to work with dressed electronic states on the dot and thus to apply a polaron transformation generating the shift *l*_0_ in the oscillator coordinate associated with a charge transfer process, i.e.,

[2]
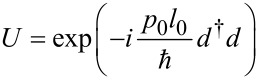


with momentum operator 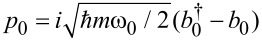 where 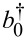 and *b*_0_ are creation and annihilation operators of the phonon mode, respectively. We mention in passing that complementary to the situation here, the theory of dynamical Coulomb blockade in ultrasmall metallic contacts is based on a transformation which generates a shift in momentum (charge) rather than position [17]. Now, the electron–phonon interaction is completely absorbed in the tunnel part of the Hamiltonian, thus capturing the cooperative effect of charge tunneling onto the dot and photon excitation in the molecule, i.e., 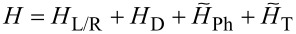 with

[3]
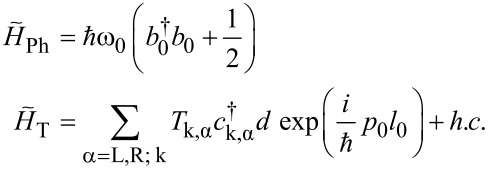


Single charge tunneling through the device can be formally and exactly captured under weak conditions (e.g., instantaneous equilibration in the leads during charge transfer) within the Meir–Wingreen formulation based on nonequilibrium Green’s functions [14–15]. For the current–voltage characteristics one finds

[4]
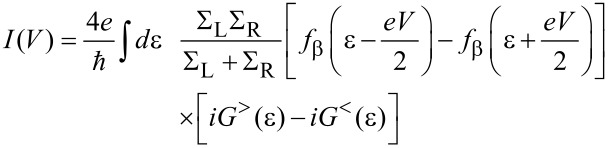


with energy dependent lead self-energies ∑_α_(ε) = 2*π*∑_k_|*T*_k,α_|^2^δ(ε – ε_k_) and with the Fourier transforms of the time dependent Green’s functions 
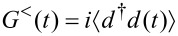
 and 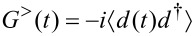. Upon applying the polaron transformation (Equation 2), one has

[5]
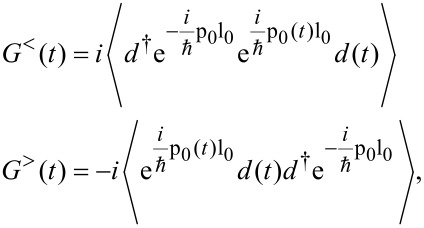


where all expectation values are calculated with the full Hamiltonian (Equation 3). Of course, for *T*_k,α_ → 0, the Green’s functions factorize as, e.g., 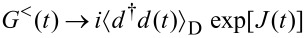 with the phonon correlation

[6]
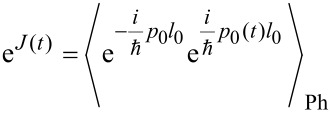


into expectation values with respect to the dot (D) and the phonon (Ph), respectively. Any finite tunnel coupling induces correlations that in analytical treatments can only be incorporated perturbatively. There, the proper approximative scheme depends on the range of parameter space one considers. Generally speaking, there are four relevant energy scales ∑_L/R_, *M*_0_, *k*_B_*T*, and 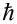
ω_0_ of the problem corresponding to three independent dimensionless parameters, e.g.,

[7]
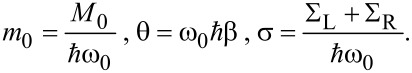


In the following we are interested in the low temperature domain *θ* > 1 where thermal broadening of phonon levels is small such that discrete steps appear in the *I*–*V* characteristics. Qualitatively, seen from the dynamics of the phonon mode, two regimes can be distinguished according to the adiabaticity parameter ∑/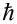
ω_0_ = σ: For σ < 1 the phonon wave packet fulfills, on a *given surface V*_0_ or *V*_1_, multiple oscillations before a charge transfer process occurs. The electron carries excess energy due to the finite voltage, and this energy may be absorbed by the phonon to promote reorganization to the new conformation (in the classical case the reorganization energy Λ). In the language of intramolecular charge transfer this scenario corresponds to the diabatic regime with well-defined surfaces *V*_q_. In the opposite regime σ > 1 charge transfer is fast such that the phonon may undergo multiple switchings *between the surfaces V*_0,1_. This is the adiabatic regime. In this latter range the impact of the adiabaticity on the diabatic ground state wave functions is weak for *m*_0_ < 1 when the distance of the diabatic surfaces is small compared to the widths of the ground states. For *m*_0_ > 1 in both regimes electron transfer is accompanied by phonon tunneling through energy barriers separating the minima of adiabatic or diabatic surfaces. The dynamics of the total compound are then determined by voltage driven, collective tunneling processes. Master equation approaches to be investigated below, rely on the assumption that both sub-units, charge degree of freedom and phonon mode preserve their bare physical properties even in the case of finite coupling *m*_0_. Hence, since the model (Equation 1) can be solved exactly in the limits *m*_0_ = 0 and σ = 0 and following the above discussion, we expect them to capture the essential physics quantitatively in the domain *m*_0_ < 1 and for all ratios σ. We note that recently the strong coupling limit including the current statistics has been addressed as well [35–36].

### 2 Rate approach I

The simplest perturbative approach considers the cooperative effect of electron tunneling and phonon excitation in terms of Fermi’s golden rule for the tunneling part 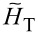. For this purpose one derives transition rates for sequential transfer according to Figure 1. A straightforward calculation for energy independent self-energies ∑_L/R_ (wide band limit) gives the forward rate onto the dot from the left lead

[8]
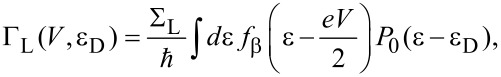


where *f*_β_(ε) is the Fermi distribution. Inelastic tunneling associated with energy emission/absorption of phonons is captured by the Fourier transform of the phonon–phonon correlation exp[*J*(*t*)] leading to

[9]
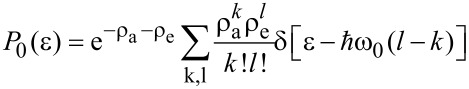


with 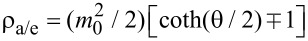 denoting the mean values for single phonon absorption (a) and emission (e). The exponentials in the prefactor contain the dimensionless reorganization energy 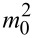 = Λ/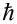
ω_0_. Apparently, inelastic charge transfer includes the exchange of multiple phonon quanta according to a Poissonian distribution. Further, one has the detailed balance relation *P*_0_(−ε) = e^−βε^*P*_0_(ε). For vanishing phonon–electron coupling *m*_0_ → 0 only the elastic peak survives, thus *P*_0_(ε) → δ(ε). We note again the close analogy to the *P*(*E*) theory for dynamical Coulomb blockade [17]. Moreover, golden rule rates for intramolecular electron transfer between donor and acceptor sites coupled to a single phonon mode are of the same form with the notable difference, of course, that in this case one has a discrete density of states for both sites [20,22]. The fundamental assumption underlying the golden rule treatment is that equilibration of the phonon mode occurs much faster than charge transfer. In the last two cases this is typically guaranteed by the presence of a macroscopic heat bath (secondary bath) strongly coupled to the prominent phonon mode. Here, the fermionic reservoirs in the leads impose phonon relaxation due to charge transfer only. Thus, for finite voltage the steady state is always a nonequilibrium state that can only roughly be described by a thermal distribution of the bare phonon system (see below). One way to remedy this problem is to introduce a phonon–secondary bath interaction as well (see below in subsection 4). The remaining transition rates easily follow due to symmetry

[10]
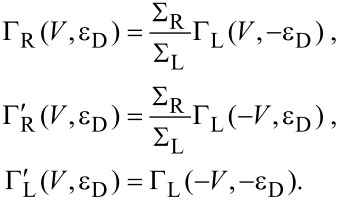


Now, summing up forward and backward events, the dot population follows from

[11]
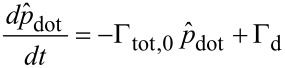


with the total rate Γ_tot,0_ = Γ_L_ + Γ_R_ + Γ'_L_ + Γ'_R_ and the rate for transfer towards the dot Γ_D_ = Γ_L_ + Γ'_R_ obtained according to Equation 8. Note that for vanishing electron–phonon coupling *M*_0_ = 0 one has 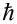
Γ_tot,0_(*M*_0_ = 0) = ∑_L_ + ∑_R_. The steady state distribution 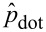 → *p*_dot_ = Γ_D_/Γ_tot,0_ is approached with relaxation rate Γ_tot,0_. For a symmetric situation ∑_L_ = ∑_R_ with ε_D_ = 0 one shows that *p*_dot_ = 1/2 independent of the voltage, while asymmetric cases lead to voltage dependent stationary populations. The steady state current is given by *I*(*V*) = (*e*/2)[(Γ_L_ – Γ'_R_)(1 – *p*_dot_) – (Γ'_L_ – Γ_R_)*p*_dot_] such that

[12]
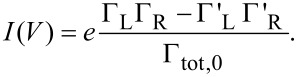


A transparent expression is obtained for ε_D_ = 0, namely,

[13]
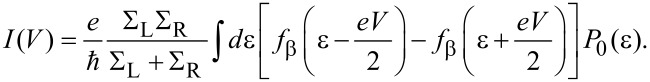


**Figure 2:**
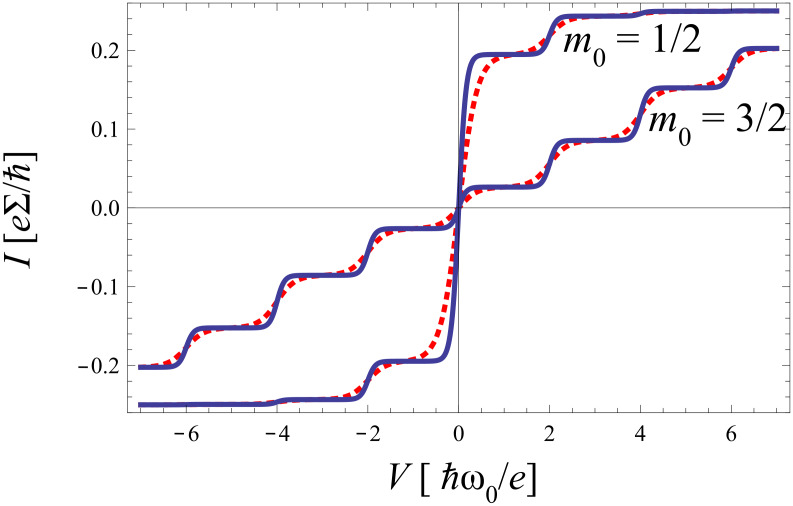
*I*–*V*-characteristics for symmetric coupling ∑_L_ = ∑_R_ and for varying electron–phonon coupling *m*_0_ at inverse temperature *θ* = 25 (solid) and *θ* = 10 (dashed).

Despite its deficiencies mentioned above, the golden rule treatment provides already a qualitative insight into the transport characteristics. Typical results are shown in Figure 2.

The *I*–*V* curves display the expected steps at 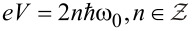. Each time the voltage *eV*/2 exceeds multiples of 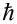
ω_0_ new transport channels open associated with the excitation of one additional phonon. For higher temperatures the steps are smeared out by thermal fluctuations. The range of validity of this description follows from the fact that a factorizing assumption for the electron–phonon correlation *and* an instantaneous equilibration of the phonon mode after a charge transfer has been used, which means that σ < 1 and *m*_0_ < 1. The latter constraint guarantees that conformational changes of the phonon distribution remain small.

There are now three ways to go beyond this golden rule approximation. With respect to the phonon mode, one way is to explicitly account for the nonequilibrium dynamics, another is to introduce a direct interaction with a secondary heat bath in order to induce sufficiently fast equilibration. With respect to the dot degree of freedom one can exploit the fact that for vanishing charge–phonon coupling the model can be solved exactly.

### 3 Master equation for nonequilibrated phonons

To derive an equation of motion for the combined dynamics of charge and phonon degrees of freedom, one starts from a Liouville–von Neumann equation for the full polaron transformed compound (Equation 3). Then, applying a Born–Markov type of approximation with respect to the tunnel coupling to the fermionic reservoirs, one arrives at a Redfield-type equation for the reduced density matrix of the dot–phonon system [15]. An additional rotating wave approximation (RWA) separates the dynamics of diagonal (populations) and off-diagonal (coherences) elements of the reduced density. Denoting with 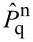 the probability to find *q* charges on the dot (here, for single charge transfer *q* = 0,1) and the phonon in its *n*-th eigenstate, one has

[14]
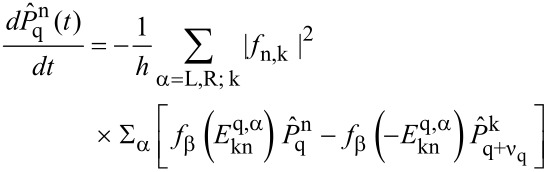


with ν_0_ = 1, ν_1_ = −1 and energies 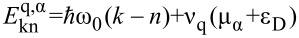. The matrix elements of the phonon shift operator 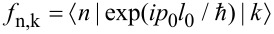 read

[15]
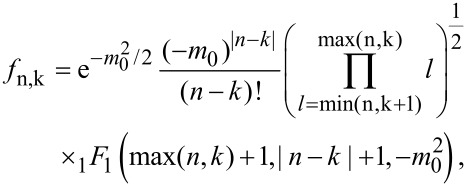


where _1_*F*_1_ denotes a hypergeometric function. The underlying assumptions of this formulation require weak dot–lead coupling σ < 1 and sufficiently elevated temperatures σ*θ* < 1 for a Markov approximation to be valid. Although we will see below when comparing low temperature results with numerically exact solutions that this seems to be only a weak constraint.

**Figure 3:**
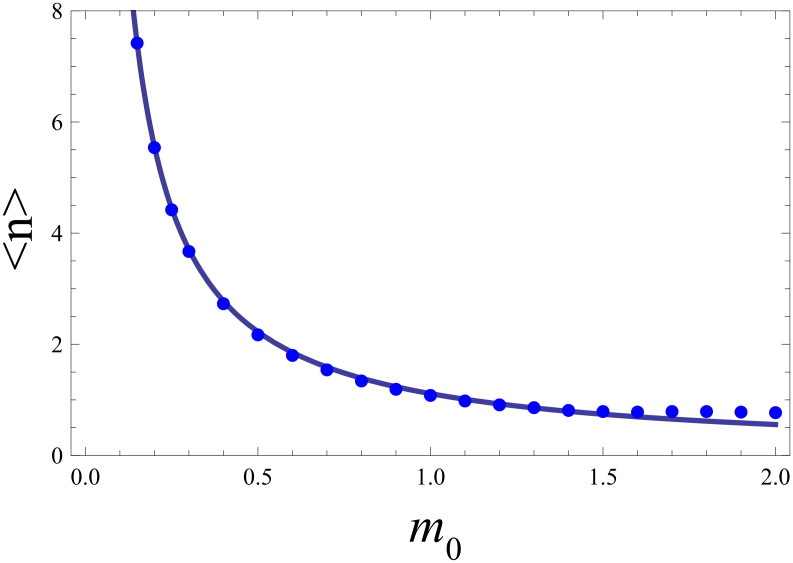
Mean phonon number in nonequilibrium for *eV* = 3
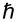
ω_0_ and versus the electron–phonon coupling *m*_0_.

The calculation of the steady state distribution 
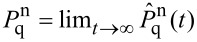
 reduces to a standard matrix inversion. One can show that for a symmetric system with ε_D_ = 0, ∑_L_ = ∑_R_ one has 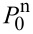 = 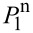. A typical example for the mean phonon number 
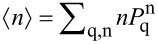
 is depicted in Figure 3. The curve is well approximated by *a*/*m*_0_ with *a* ≈ 0.7. Apparently, <*n>* diverges for *m*_0_ → 0 since then 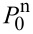 and 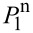 approach constants independent of the phonon number. Upon closer inspection one finds that excitation is more likely than absorption, i.e., *f* (*n*,*n* + 1) > *f* (*n*,*n* − 1), for all 0 ≤ *n* ≤ *N*_0_(*m*_0_) where *N*_0_(*m*_0_) increases with decreasing *m*_0_. The opposite is true for *n* > *N*_0_(*m*_0_) such that in a steady state, depending on the voltage, the tendency is to have higher excited phonon states occupied by smaller couplings *m*_0_. In particular, for strong coupling transitions *n* → *n* + *k*,*k* ≥ 0 are blocked at small *n*.

**Figure 4:**
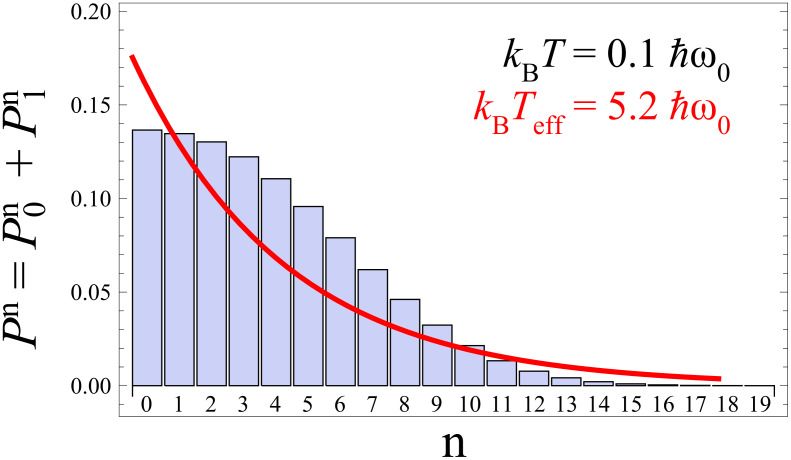
Phonon number distribution in nonequilibrium for *eV* = 5
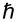
ω_0_, *m*_0_ = 0.5 and *k*_B_*T*/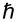
ω_0_ = 0.1 (histogram). The solid line depicts a fit to a Boltzmann distribution. See text for details.

A convenient strategy to include nonequilibrium effects in the phonon distribution, sometimes used in the interpretation of experimental data, is the introduction of an effective temperature *T*_eff_. This way one could return to the simpler modeling of the previous section. However, the procedure to identify 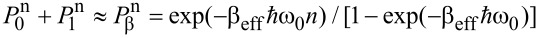 is not reliable, as Figure 4 reveals. While it clearly shows the general tendency of a substantial heating of the phonon degree of freedom induced by the electron transfer, the profile of a thermal distribution strongly differs from the actual steady state distribution.

Nonequilibrated phonons leave their signatures also in the *I*–*V* curves as compared to equilibrated ones. The net current through the contact follows from the summing up of the transfer rates from/onto the dot according to Equation 14, hence,

[16]
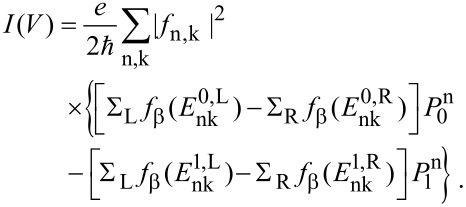


**Figure 5:**
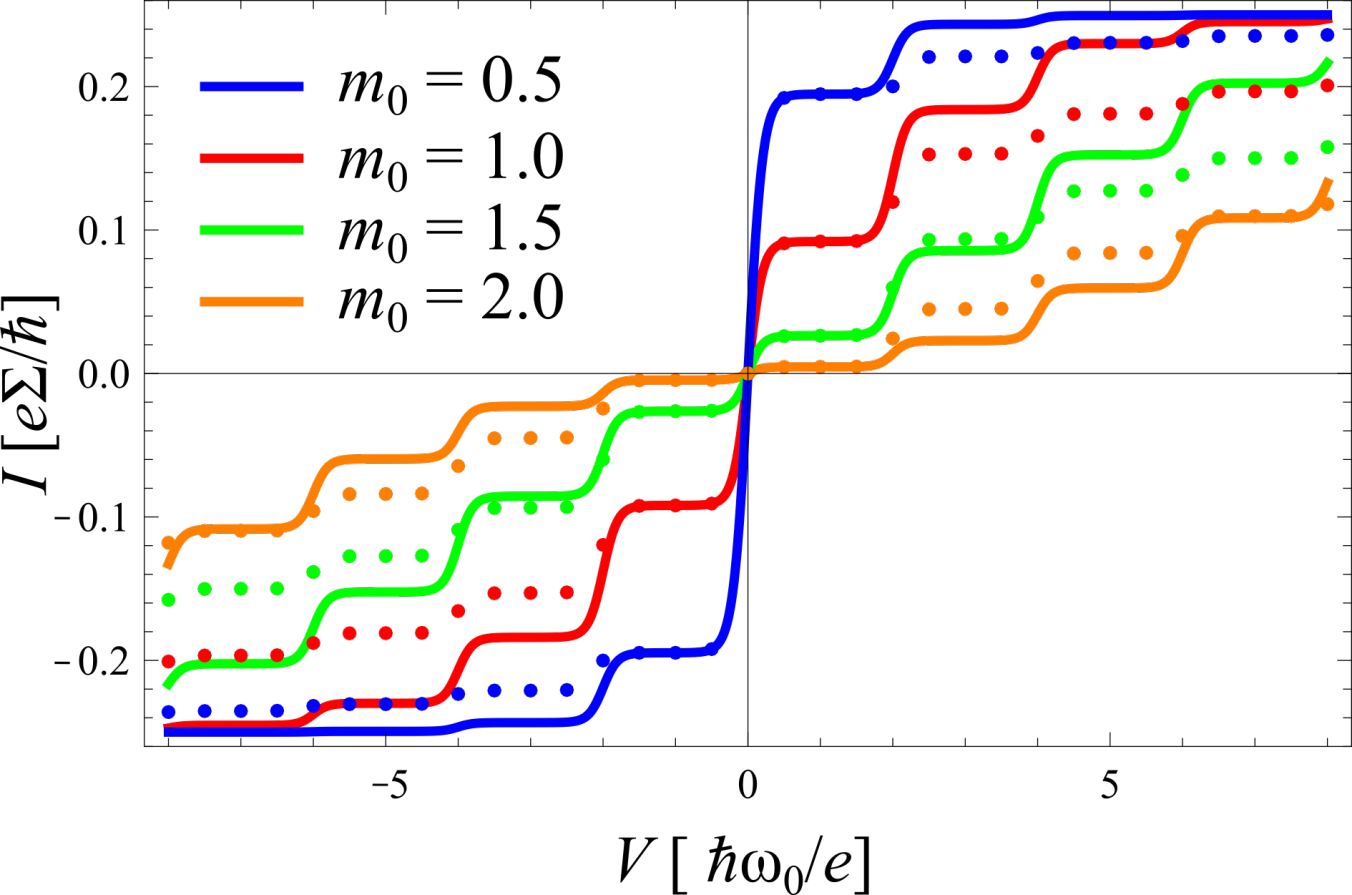
*I*–*V*-characteristics for equilibrated (solid) and nonequilibrated (dotted) phonon distributions according to Equation 13 and Equation 16, respectively.

Figure 5 shows that deviations are negligible for low voltages in the regime around the first resonant step (|*eV*/2| < 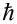
ω_0_), where at sufficiently low temperatures only the ground state participates such that the steady state distribution coincides with the thermal one. For larger voltages deviations occur with the tendency that for smaller couplings *m*_0_ the nonequilibrated current is always smaller than the equilibrated one (*I*_non_ < *I*_eq_), while the opposite scenario (*I*_non_ > *I*_eq_) is observed for larger *m*_0_. At sufficiently large voltages, one always has *I*_non_ < *I*_eq_. This behavior results from the combination of two ingredients, namely, the phonon distributions 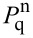 and the Franck–Condon overlaps |*f*_n,k_|^2^. To see this in detail, let us consider a fixed voltage. Then, on the one hand, for smaller *m*_0_ the steady state distribution is broad (cf. Figure 3), such that, due to normalization, less weight is carried by lower lying states compared to a thermal distribution at low temperatures; on the other hand, for *m*_0_ < 1 the overlaps |*f*_n,k_|^2^ favor contributions from low lying states in the current (16), which is thus smaller than *I*_eq_. For increasing electron–phonon coupling *m*_0_ > 1, the overlaps |*f*_n,k_|^2^ tend to include broader ranges of phonon states also covered by 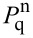, compared to those of low temperature thermal states. A voltage dependence arises since with increasing voltage higher lying phonon states participate in the dynamics supporting the scenario for smaller couplings. Interestingly, as already noted in [23] the overlaps |*f*_n,k_|^2^ may vanish for certain combinations of *n*,*m* depending on *m*_0_ due to interferences of phonon eigenfunctions localized on different diabatic surfaces *V*_q_, where *q* = 0,1.

### 4 Rate approach II

The assumption of a thermally distributed phonon degree of freedom during the transport can be physically justified only if this mode interacts directly and sufficiently strongly with an additional heat bath (secondary bath) realized, e.g., by residual molecular modes. Here we will generalize the formulation of subsection 2 to a situation where the secondary bath is characterized by Gaussian fluctuations. Its corresponding modes can thus effectively be represented by a quasi-continuum of harmonic oscillators for which the phonon correlation function (Equation 6) can be calculated easily

[17]
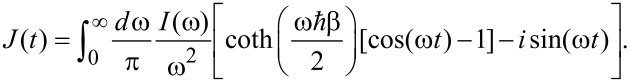


Here the spectral density *I*(ω) now describes the combined distribution of the prominent mode and its secondary bath. It is thus proportional to the imaginary part of the dynamic susceptibility of a damped harmonic oscillator [20]. For a purely ohmic distribution of bath modes, one has

[18]
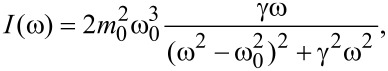


where γ denotes the coupling between phonon mode and bath. The Fourier transform of exp(*J*) reads at finite temperatures

[19]
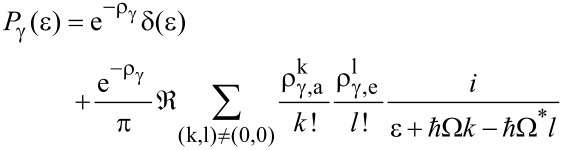


with the frequency Ω given by Ω = ω_0_ξ + *i*γ/2 and 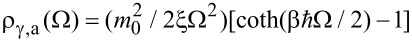 where the parameter ξ is 
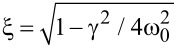
. Further, ρ_γ,_*_e_*(Ω) = −ρ_γ,_*_e_*(Ω^*^) (* means complex conjugation) and ρ_γ_ = 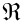
[ρ_γ,_*_a_* + ρ_γ,_*_e_*]/2. In the above expression, contributions from the Matsubara frequencies in Equation 17 have been neglected, since they are only relevant in the regime γ
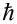
β >> 2π, which is not studied here. Apparently, the coupling to the bosonic bath effectively induces a broadening of the dot levels 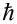
γ(*k* + *l*)/2 compared to the purely elastic case (Equation 9). In the low temperature regime, where for equilibrated phonons absorption (related to *k*) is negligible, the widths grow proportionally to *l*. The presence of the secondary bath drives the prominent phonon mode towards thermal equilibrium with a rate proportional to this broadening. Hence, if the time scale for thermal relaxation is sufficiently smaller than the time scale for charge transfer, i.e., 1/τ_l_ ≡ (∑_L_ + ∑_R_)/γ << 1, the assumption of an equilibrated phonon mode is justified and the golden rule formulation (Equation 13) can be used with *P*_0_(ε) → *P*_γ_(ε). However, this argument no longer applies in the overdamped situation γ/ω_0_ >> 1, where the phonon mode exhibits a sluggish thermalization on the time scale 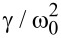, which may easily exceed τ_l_.

As already mentioned above, for vanishing charge–phonon coupling *m*_0_ = 0, the model (Equation 3) can be solved exactly for all orders in the lead–dot coupling [15]. In the frame of a rate description, one observes that in this limit the dot population (Equation 11) decays proportionally to (∑_L_ + ∑_R_). The golden rule version of the theory neglects this broadening in Equation 13 since it is associated with higher order contributions to the current (Equation 13). Now, recalling that *P*_0_(ε) reduces to a delta function for *m*_0_ → 0, this finite lifetime of the electronic dot level is included for *all orders* by performing the time integral in the Fourier transform with ε → ε − *i*(∑_L_ + ∑_R_)/2 ≡ ε − *i*Γ_tot_(*M*_0_ = 0)/2 [see Equation 11]. In fact, this way one reproduces the *exact* solution (one electronic level coupled to leads with energy independent couplings), i.e., its exact spectral function. To be specific, let us restrict ourselves for the remainder of this discussion to the symmetric situation ∑_L_ = ∑_R_ ≡ ∑/2, and ε_D_ = 0. Then, in the presence of the phonon mode (*m*_0_≠ 0) the corresponding function 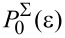 follows from Equation 9 by replacing the delta function by *i*/[ε + 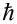
ω(*k* − *l*) + *i*∑/2]. Again following the idea of a rate treatment, an improved version of this result accounting for higher order electron–phonon correlations is obtained through the decay rate Γ_tot,0_(*M*_0_ ≠ 0), instead of the bare dot level width ∑/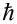 ≡ Γ_tot,0_(*M*_0_ = 0). Equivalently, one replaces *i*/[ε + 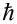
ω(*k* − *l*) + *i*∑/2] → *i*/[ε + 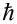
ω(*k* − *l*) + *i*Γ_tot,0_/2] to arrive at an improved 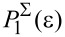. We note that within a Green’s function approach, and upon approximating the corresponding equations of motion, a similar result has been found in [15,33], with the difference though that instead of Γ_tot,0_ an imaginary part of a phonon state-dependent self-energy 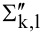 appears. One can show that the Γ_tot,0_ appearing here within a rate scheme is related to a thermally averaged 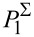.

Now, an additional secondary bath can be introduced as above by combining Equation 19 with 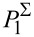, leading eventually to

[20]
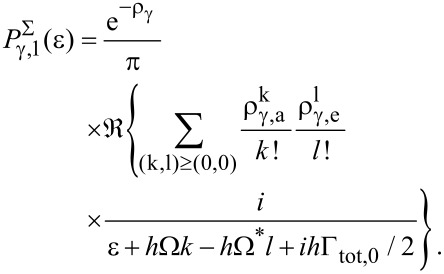


The width of the electronic dot level is thus voltage dependent and approaches the bare width from below for large voltages, that is lim*_V_*_→∞_Γ_tot,0_(*V*) = ∑/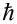. The range of validity of this scheme is the following: It applies to all couplings σ in the domain where the electron–phonon coupling is weak *m*_0_ < 1. In particular, second order processes in σ capture cotunneling processes. For *m*_0_ > 1 charge transfer is strongly suppressed and the phonon dynamics still occurs on diabatic surfaces for σ << 1 so that we expect the approach to cover this range as well.

### 5 Comparison with numerically exact results

A numerically exact treatment of the nonequilibrium dynamics of the model considered here is a formidable task. The number of formulations which allow simulations in nonperturbative ranges of parameter space is very limited. Among them is a recently developed diagrammatic Monte Carlo approach (diagMC) based on a numerical evaluation of the full Dyson series, which, in contrast to numerical renormalization group (NRG) methods [37], covers the full temperature range. For calculations of single charge transfer, results have been obtained with and without the presence of a secondary bath interacting with the dot phonon mode.

We note that computationally these simulations are very demanding as for each parameter set and a given voltage the stationary current for the *I*–*V* curve needs to be extracted from the saturated value of the time dependent current *I*(*t*) for longer times. Typical simulation times are on the order of several days to weeks, depending on the parameter range. In contrast, rate treatments require minimal computational effort and can be done within minutes. Here, we compare numerically exact findings with those gained from the various types of rate/master equations discussed above.

We start with the scenario where the coupling to a secondary bath is dropped (γ = 0) to reveal the impact of nonequilibrium effects in the phonon mode. The formulation for an equilibrated phonon is based on Equation 13 with *P*_0_ replaced by 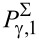 in Equation 20, while the steady state phonon distribution is obtained from the stationary solutions to Equation 14. In the latter approach the intrinsic broadening of the dot electronic level due to coupling to the lead is introduced in the following way: One first determines via Equation 14 a steady state distribution 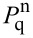. This result is used for an effective self-energy contribution (total decay rate) for nonequilibrated phonons, i.e.,

[21]
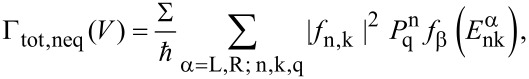


where 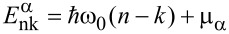. We note in passing that lim*_V_*_→∞_Γ_tot,neq_(*V*) = ∑/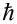 ≡ Γ_tot_(*M*_0_ = 0). Subsequently, an improved result for the steady state phonon distribution at a given voltage is evaluated working again with Equation 14, but using the replacement:

[22]
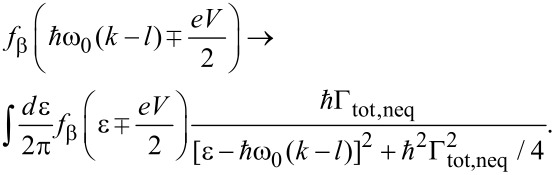


Of course, for ∑ → 0 the standard Fermi distribution is regained. The corresponding steady state phonon distribution eventually provides the current according to Equation 16 with the same replacement (Equation 22) in this expression. The procedure relies on weak electron–phonon coupling *m*_0_ < 1 and in principle also requires sufficiently elevated temperatures.

**Figure 6:**
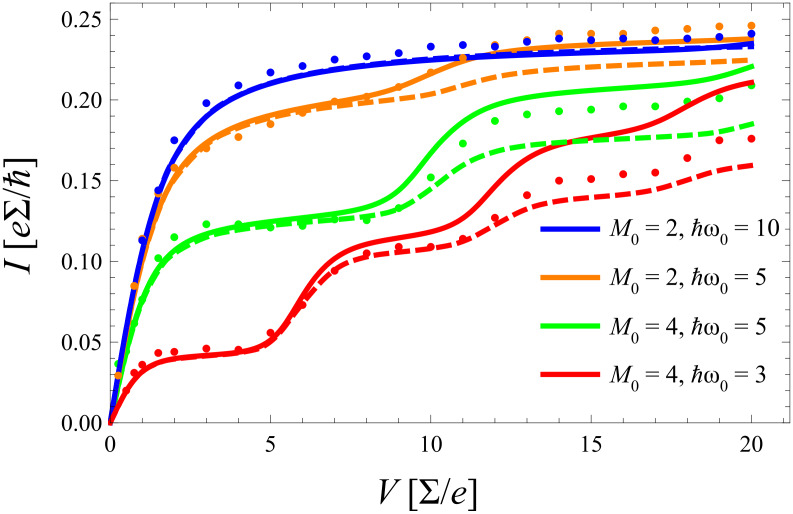
*I*–*V* characteristics according to approximate models based on equilibrated phonons (solid) and nonequilibrated phonons (dashed) together with exact DQMC data (dots) for *k*_B_*T*/∑ = 0.2 and without coupling to a secondary bath (γ = 0). All quantities are scaled with respect to the dot–lead coupling ∑.

Results are shown in Figure 6 together with corresponding diagMC data for various coupling strengths *m*_0_. Interestingly, the equilibrated model describes the exact data very accurately from weak up to moderate electron–phonon coupling *m*_0_ ≈ 1, while deviations appear for stronger couplings 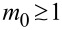 and voltages beyond the first plateau *eV* > 2
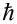
ω_0_. For *m*_0_ > 1 nonequilibrium effects are stronger and the corresponding master equation (Equation 14) gives a better description of higher order resonant steps. Moreover, as already addressed above, even in this low temperature domain the approximate description provides quantitatively reliable results.

**Figure 7:**
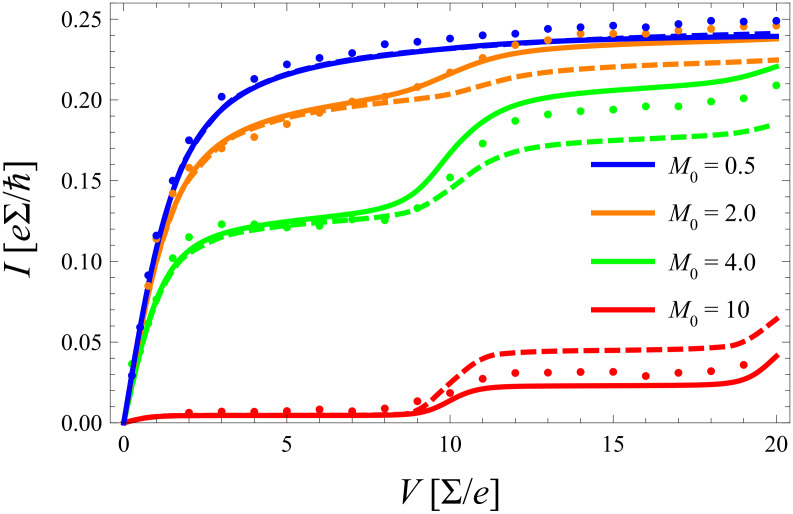
As Figure 6 but for fixed 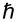
ω_0_/∑ = 5 and varying electron–phonon coupling.

In Figure 7 the frequency of the phonon mode is fixed and only the electron–phonon coupling is tuned over a wider range. For strong coupling (here *m*_0_ = 2) the equilibrated (nonequilibrated) model predicts a smaller (larger) current than the exact one in contrast to the situation for smaller *m*_0_. This phenomenon directly results from what has been said above in subsection 3: For stronger coupling the Franck–Condon overlaps favor higher lying phonon states that are suppressed by a thermal distribution.

After all, the approximate models give not only a qualitatively correct picture of the exact *I*–*V* curves, but even provide a reasonable quantitative description in this low temperature domain.

**Figure 8:**
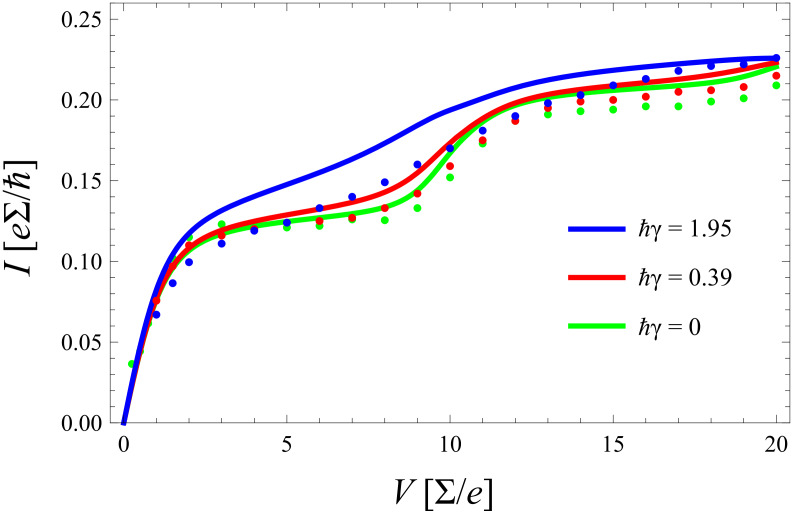
*I*–*V* characteristics in presence of a secondary heat bath interacting with the phonon with various coupling constants γ. Shown are approximate results (solid) using Equation 20 and diagMC data (dots); energies are scaled with ∑. Other parameters are *k*_B_*T*/∑ = 0.2, *m*_0_ = 4/5, σ = 0.2.

In a next step the coupling to a secondary bath is turned on (γ ≠ 0) enforcing equilibration of the phonon mode, see Equation 20. The expectation is that in this case departures from the equilibrated model are reduced. In Figure 8 data are shown for a ratio *m*_0_ = 4/5, where deviations occur at larger voltages, as observed in the previous figures. Obviously, due to the damping of the phonon mode the resonant steps are smeared out with increasing γ. However, the approximate model predicts this effect to be more pronounced as compared to the exact data, particularly for stronger coupling 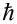
γ/∑ > 1, while still γ/ω_0_ < 1. In fact, in the limit of very large coupling only the *k* = *l* = 0 contribution to Equation 20 survives, such that at zero temperature one arrives at

[23]
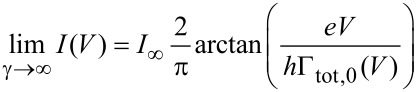


with the current at large voltages *I*_∞_ = *e*∑/4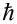 and Γ_tot,0_(*V*) ≤ ∑/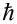, where equality is approached for *V* → ∞. It seems that a broadened equilibrium distribution of the phonon, induced by the secondary bath according to Equation 20, overestimates the broadening of individual levels. Since the approach is exact in the limit *m*_0_ → 0, the deviations appearing in Figure 8 are due to intimate electron–phonon/secondary bath correlations not captured by the rate approach. In the overdamped regime, i.e., γ/ω_0_ > 1, the dynamics of the phonon mode slow down and may become almost static on the time scale of the charge transfer. In this adiabatic regime an extended version of the master equation (Equation 14) is not trivial since the conventional eigenstate representation becomes meaningless. It would be better then to switch to phase-space coordinates and develop a formulation based on a Fokker–Planck or Smoluchowski equation for the phonon. This will be the subject of future research.

**Figure 9:**
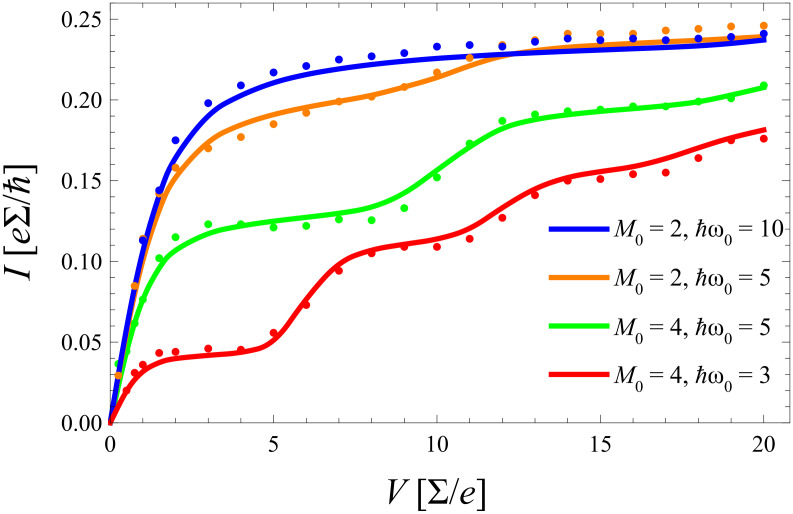
As Figure 6, but for nonequilibrated phonons based on an extended master equation (solid) in comparison to exact diagMC data (dots).

**Figure 10:**
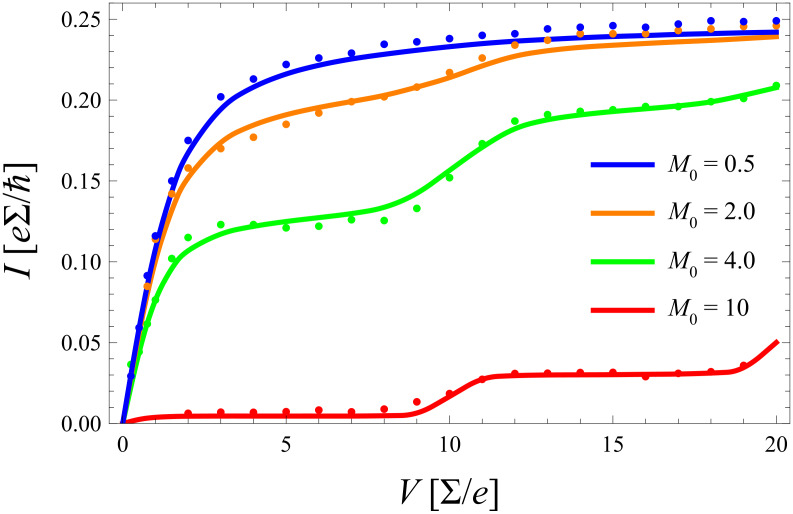
As Figure 7, but for nonequilibrated phonons based on an extended master equation (solid) in comparison to exact diagMC data (dots).

The essence of this comparison is that, as anticipated from physical arguments already in subsection 1, a rate description does indeed provide quantitatively accurate results in the regime of weak to moderate electron–phonon coupling *m*_0_ < 1 and for all σ. Deviations that occur for larger values of *m*_0_ can partially be explained by nonequilibrium distributions in the phonon distribution, where, however, the master equation approach seems to overestimate this effect. In order to obtain some insight into the nature of this deficiency, a minimal approach consists of extending Equation 14 with Equation 22, a mechanism that enforces relaxation to thermal equilibrium with a single rate constant Γ_0_ that serves as a fitting parameter. Accordingly, the respective time evolution equation for 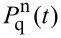 receives an additional term −Γ_0_[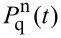 − 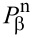] with the Boltzmann distribution for the bare phonon degree of freedom 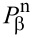. Corresponding results for the same parameter range as in Figure 6 and Figure 7 are shown in Figure 9 and Figure 10 including comparison with the exact diagMC data. There, the *same* equilibration rate 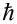
Γ_0_/∑ = 0.25 is used for *all* parameter sets. Astonishingly, this procedure provides excellent agreement over the full voltage range. It improves results particularly in the range of moderate to stronger electron phonon coupling, but has only minor impact for *m*_0_ < 1. The indication is thus that electron–phonon correlations neglected in the original form of the master equation have effectively the tendency to support faster thermalization of the phonon. Indeed, preliminary results with a generalized master equation, where the coupling between diagonal (populations) and off-diagonal (coherences) elements of the reduced charge–phonon density matrix is retained (no RWA approximation), indicate that this coupling leads to an enhanced phonon–lead interaction and thus to enhanced phonon equilibration.
